# Signal recovery from stimulation artifacts in intracranial recordings with dictionary learning

**DOI:** 10.1088/1741-2552/ab7a4f

**Published:** 2020-04-09

**Authors:** D J Caldwell, J A Cronin, R P N Rao, K L Collins, K E Weaver, A L Ko, J G Ojemann, J N Kutz, B W Brunton

**Affiliations:** 1 Department of Bioengineering, University of Washington, Seattle, WA, United States of America; 2 Medical Scientist Training Program, University of Washington, Seattle, WA, United States of America; 3 Center for Neurotechnology, Seattle, WA, United States of America; 4 University of Washington Institute for Neuroengineering, Seattle, WA, United States of America; 5 Paul G. Allen School of Computer Science and Engineering, University of Washington, Seattle, WA, United States of America; 6 Department of Radiology, University of Washington, Seattle, WA, United States of America; 7 Department of Neurological Surgery, University of Washington, Seattle, WA, United States of America; 8 Department of Applied Mathematics, University of Washington, Seattle, WA, United States of America; 9 Department of Biology, University of Washington, Seattle, WA, United States of America; 10 Author to whom any correspondence should be addressed.

**Keywords:** electrophysiology, stimulation, artifact, neural signals

## Abstract

**Objective.:**

Electrical stimulation of the human brain is commonly used for eliciting and inhibiting neural activity for clinical diagnostics, modifying abnormal neural circuit function for therapeutics, and interrogating cortical connectivity. However, recording electrical signals with concurrent stimulation results in dominant electrical artifacts that mask the neural signals of interest. Here we develop a method to reproducibly and robustly recover neural activity during concurrent stimulation. We concentrate on signal recovery across an array of electrodes without channel-wise fine-tuning of the algorithm. Our goal includes signal recovery with trains of stimulation pulses, since repeated, high-frequency pulses are often required to induce desired effects in both therapeutic and research domains. We have made all of our code and data publicly available.

**Approach.:**

We developed an algorithm that automatically detects templates of artifacts across many channels of recording, creating a dictionary of learned templates using unsupervised clustering. The artifact template that best matches each individual artifact pulse is subtracted to recover the underlying activity. To assess the success of our method, we focus on whether it extracts physiologically interpretable signals from real recordings.

**Main results.:**

We demonstrate our signal recovery approach on invasive electrophysiologic recordings from human subjects during stimulation. We show the recovery of meaningful neural signatures in both electrocorticographic (ECoG) arrays and deep brain stimulation (DBS) recordings. In addition, we compared cortical responses induced by the stimulation of primary somatosensory (S1) by natural peripheral touch, as well as motor cortex activity with and without concurrent S1 stimulation.

**Significance.:**

Our work will enable future advances in neural engineering with simultaneous stimulation and recording.

## 1. Introduction

Direct electrical stimulation of the brain is a powerful tool in both clinical and basic neuroscience; it is useful for probing neural circuitry (Matsumoto *et al* 2004, Keller *et al* 2014), modifying cortical connections (Zanos *et al* 2018, Keller *et al* 2018), as well as providing direct feedback to brain regions (Caldwell *et al* 2019, Cronin *et al* 2016, Collins *et al* 2017, Lee *et al* 2018, Hiremath *et al* 2017, Flesher *et al* 2017). Importantly, electrical stimulation has the potential to advance the emerging field of neuroprosthetics (Caldwell *et al* 2019). However, the interpretation of neural activity during concurrent electrical stimulation is a substantial challenge. The recorded stimulus artifact induced by electrical stimulation is often orders-of-magnitude greater than the neural signals of interest, confounding traditional analytic techniques, such as time-frequency and time-series analyses (Zhou *et al* 2018). Both hardware and analytic approaches have been developed to minimize the impact of electrical artifacts on neural recordings. Emerging front-end hardware solutions are promising (Zhou *et al* 2018, Weiss *et al* 2019), but they will likely continue to work in concert with further processing after signal acquisition.

There is a wealth of work in the literature developing computational methodology to process recordings with concurrent electrical stimulation. These methods can be summarized as taking one of five different approaches to manage the stimulation artifact: *simple interpolation*, *template subtraction*, *biophysical modeling of the artifact*, *frequency-based filtering of the signal*, and *modal decomposition of the artifact from the signal*. Fortunately, an increasing number of these methods have been made available through toolboxes with code and data for rapid dissemination and evaluation of the results (Erez *et al* 2010, Herring *et al* 2015). Interpolation and curve fitting through the artifact window is a simple but often effective approach for short-duration artifacts, especially when the neural responses during these short artifacts are not the focus of the study (Wagenaar *et al* 2002, Heffer *et al* 2008, Voigt *et al* 2018). Beyond interpolation, a variety of methods have instead subtracted an averaged template of the artifact window to estimate the underlying signal (Hashimoto *et al* 2002, Sun *et al* 2016). Interestingly, these approaches have been extended for proposed real-time closed-loop stimulation, with validation on simulated ECoG data with real stimulation artifacts (Yang *et al* 2018). The template extraction method has been extended with a variation using unsupervised manifold learning (Alagapan *et al* 2018). An alternative approach is to make simplifying assumptions and construct biophysical models of the interactions between the electrical stimulation and the tissue to isolate the neural signals (Walker *et al* 2012, Trebaul *et al* 2016). However, it is challenging to balance adequate recovery of the neural signal with complete artifact elimination in these time-domain subtraction methods.

The stimulation artifact can also be processed in the frequency domain. A variety of filters have been developed to match the characteristics of the artifact, including band-stop filters (Jech *et al* 2006, Sun *et al* 2014), a Hampel filter (Allen *et al* 2010), and a recursive Wiener filter (Mouthaan *et al* 2016), to name a few. Even with these advanced filtering methods, it remains difficult to isolate the neural signal when the frequency spectra of the signal and the artifact overlap (Nagel *et al* 2000, Lio *et al* 2018).

By leveraging correlations across multiple, simultaneously-recorded channels, much progress has been made using modal decomposition techniques to separate the neural signals from the artifacts. Commonly used methods have included independent component analysis (ICA, (Gilley *et al* 2006, Lu *et al* 2012)) and empirical mode decomposition (EMD, (Al-ani *et al* 2011)). Another recent work models the spatial and temporal correlations among a large array of electrical recordings as structured Gaussian processes (Mena *et al* 2017). Modal decomposition has also been used to generate artifact templates by sequential principal component regression (PCR), which are subsequently subtracted from the recordings (O’Shea *et al* 2017).

In this paper, we develop a data-driven method based upon dictionary learning to recover neural signals from recordings with concurrent electrical stimulation. The method proposed here is a type of template subtraction; our innovation lies in using unsupervised machine learning to learn a dictionary of artifact templates. In particular, our method is designed to perform well with ongoing trains of stimulation across many channels in human intracranial recordings. Trains of stimulation are frequently used to provide therapeutic benefit through modalities such as deep brain stimulation (DBS) (Ashkan *et al* 2017, Montgomery *et al* 2008, Montgomery *et al* 2011), responsive cortical stimulation for the treatment of epilepsy (Sohal *et al* 2011), and to provide sensory information in neuroprosthetic applications with direct electrical stimulation (DES) of primary somatosensory cortex (S1) (Cronin *et al* 2016, Lee *et al* 2018, Flesher *et al* 2017, Hiremath *et al* 2017). When many stimulation trains are used, pulses may have varying stimulation parameters, the recorded waveforms may differ depending on the sampling, and their proximity to each other may lead to ambiguity in separation from the neural signals. Our method allows for the discovery of independent clusters of artifacts across many channels, without user defined onsets and offsets. Furthermore, outlier stimulation pulses and trials may be excluded by the automatic clustering algorithm employed (HDB-SCAN), preventing the contamination of the most salient, core artifact clusters present throughout a recording.

We demonstrate the performance of our method on several different human intracranial experiments, including stimulation of S1 and DBS. For the S1 stimulations, we also compare the evoked cortical responses across the array of recording electrodes for DES and natural haptic touch. In addition, we show that our method extracts adjacent motor cortical activity with concurrent S1 stimulation. Such signal extraction is important for simultaneous motor decoding and sensory stimulation for closed-loop neuroprosthetics. Furthermore, our extraction of neural signals in response to DBS shows the utility of our method for understanding the neural response to this clinical application of electrical stimulation. We have made all of the code and data publicly available to accelerate future research in neural engineering with concurrent stimulation and recording. Our datasets include five different subjects performing five different tasks; we hope that these datasets will serve as common testbeds for others pursuing future algorithmic developments. We believe our method and work here advances the field of artifact processing in neural data due to the combination of advanced unsupervised clustering techniques, automatic artifact detection, application across many channels and with trains of closely spaced stimulation pulses, demonstrations of failure modes, and open-source code with five unique human datasets.

## 2. Methods and datasets

### 2.1. Human subjects

Human subjects with medically intractable epilepsy undergoing inpatient invasive electrophysiologic mapping of their seizure focus were implanted at Harborview Medical Center (Seattle, WA) with subdural electrocorticographic (ECoG) grids (8 by 8 contacts, 2.3 mm exposed diameter, Ad-tech Medical, Racine, WI, USA). ECoG grid placement was determined solely based on clinical needs without consideration of research benefits. Although the clinical management of each patient was tailored to their specific medical needs, the subjects’ antiepileptic medications generally were weaned or fully discontinued during the period of monitoring until a sufficient quantity of seizures had been captured to adequately localize their seizure focus, following which they resumed taking full doses of their anti-epileptic medications. We conducted all stimulation studies after subjects were back on their anti-epileptic medications, after approximately one week of clinical monitoring.

Human DBS subjects were implanted at University of Washington Medical Center (Seattle, WA) with DBS leads (Medtronic) and subdural ECoG strips (1 by 8 contacts, Ad-tech Medical, Racine, WI, USA).

All patients gave informed consent under protocols approved by the University of Washington Institutional Review Board.

### 2.2. Cortical reconstructions

We performed cortical reconstructions using previously described techniques (Blakely *et al* 2009, Hermes *et al* 2010, Wander *et al* 2016).

### 2.3. Data acquisition and stimulation

Neural data were acquired at 1221, 12 207 or 48 828 Hz using a Tucker Davis Technologies (TDT) System 3 with the RZ5D and PZ5 Neurodigitizer (Tucker Davis Technologies, Alachua, Florida, USA). Neural data were acquired from the non-stimulating electrodes. All datasets, except for the DBS dataset, were acquired with 64 contact macro ECoG grid arrays, while the DBS dataset was acquired with 4 contact cylindrical DBS leads and 8 contact macro ECoG strips. We delivered stimulation through the TDT IZ2H-16 stimulator and LZ48–400 battery pack (Tucker Davis Technologies), with the precise stimulation parameters determined by the particular experiment (detailed below). For all experiments, the stimulation pairs of electrodes were adjacent electrodes on the electrode array.

### 2.4. Haptic touch dataset

The neural data acquired for comparisons between stimulation of S1 and natural haptic touch are from previously described experiments (Caldwell *et al* 2019), in which subjects received stimulation of hand S1 somatosensory cortex and peripheral haptic touch of the corresponding region of the hand during a response timing task. Briefly, a pair of electrodes through which to apply bipolar stimulation of hand S1 was selected informed by anatomic locations of the electrodes and prior clinical mapping. Stimulation was then applied, and using verbal feedback of the subjects, a pair of electrodes which elicited reproducible percepts on the subject’s hands was selected for further sensory experiments. The haptic touch was then applied to the cutaneous area where subjects reported the sensation from stimulation of hand S1. We used 200 Hz, biphasic (200 *μ*s pulse width), bipolar, 2 mA constant current stimulation trains of 400 ms duration.

### 2.5. Non-uniform train dataset

The neural data acquired for the non-uniform train set were sampled at 12 207 Hz. Stimulation waveforms consisted of two, 3 mA pulses followed by 38,1.5 mA pulses at 200 Hz, for a total of 200 ms of bipolar, biphasic (200 *μ*s pulse width) stimulation.

### 2.6. Button press dataset

The neural data acquired during motor behavior with stimulation of sensory cortex was acquired with a subject performing a self-paced motor button pressing task with and without concurrent, random stimulation of S1. The subject was instructed to press the button firmly and consistently, while an experimenter randomly stimulated S1 during the activity. The subject was told not to respond differently based on the stimulation and was able to complete the button pressing task both with and without stimulation. We used 200 Hz, 200 *μ*s pulse width, biphasic, bipolar,1.5 mA constant current stimulation trains of 200 ms duration.

### 2.7. DBS dataset

The DBS data were acquired intraoperatively during DBS lead placement, with stimulation and recording through the TDT system on a four-contact implanted Medtronic lead. Subjects received 15 epochs of 4 different stimulation amplitudes (1.5, 2, 2.5, 3 V, all of 500 ms duration) with 185 Hz, bipolar, monophasic (60 *μ*s pulse width) constant voltage stimulation, for a total of 60 epochs.

### 2.8. Rubber hand illusion dataset

The neural data to illustrate inadequate sampling for stimulation epoch processing using the dictionary learning method were sampled at 1221 Hz, as previously described in (Collins *et al* 2017). 100 Hz,2.2 mA constant current, biphasic (200 *μ*s pulse width), bipolar stimulation was used.

## 3. Signal recovery algorithm

Our goal was to develop an algorithmic approach to recover neural signals recorded concurrently with electrical stimulation. We have designed our algorithm based on three key assumptions about the neural data. First, stimulation artifacts are assumed to be additive and do not interact nonlinearly with the signal, so that once estimated, they can be subtracted from the recording. Second, we assume that the data are acquired by recording amplifiers that do not saturate during stimulation, are capable of oversampling, and are synchronized between recording and stimulation devices. Third, we assume that the timing of artifact windows may be extracted in all channels by the stimulation onset from a single channel; this assumption draws on knowledge that volume conduction is much faster than neural signal propagation. Our software includes options to recover stimulation-evoked pulses, and to dynamically detect the offset of stimuli pulses on a channel and pulse-wise basis. In our online code repository and associated readme file, we discuss the meanings of various parameters, as well as guidance in selecting their values.

**Figure 1.**
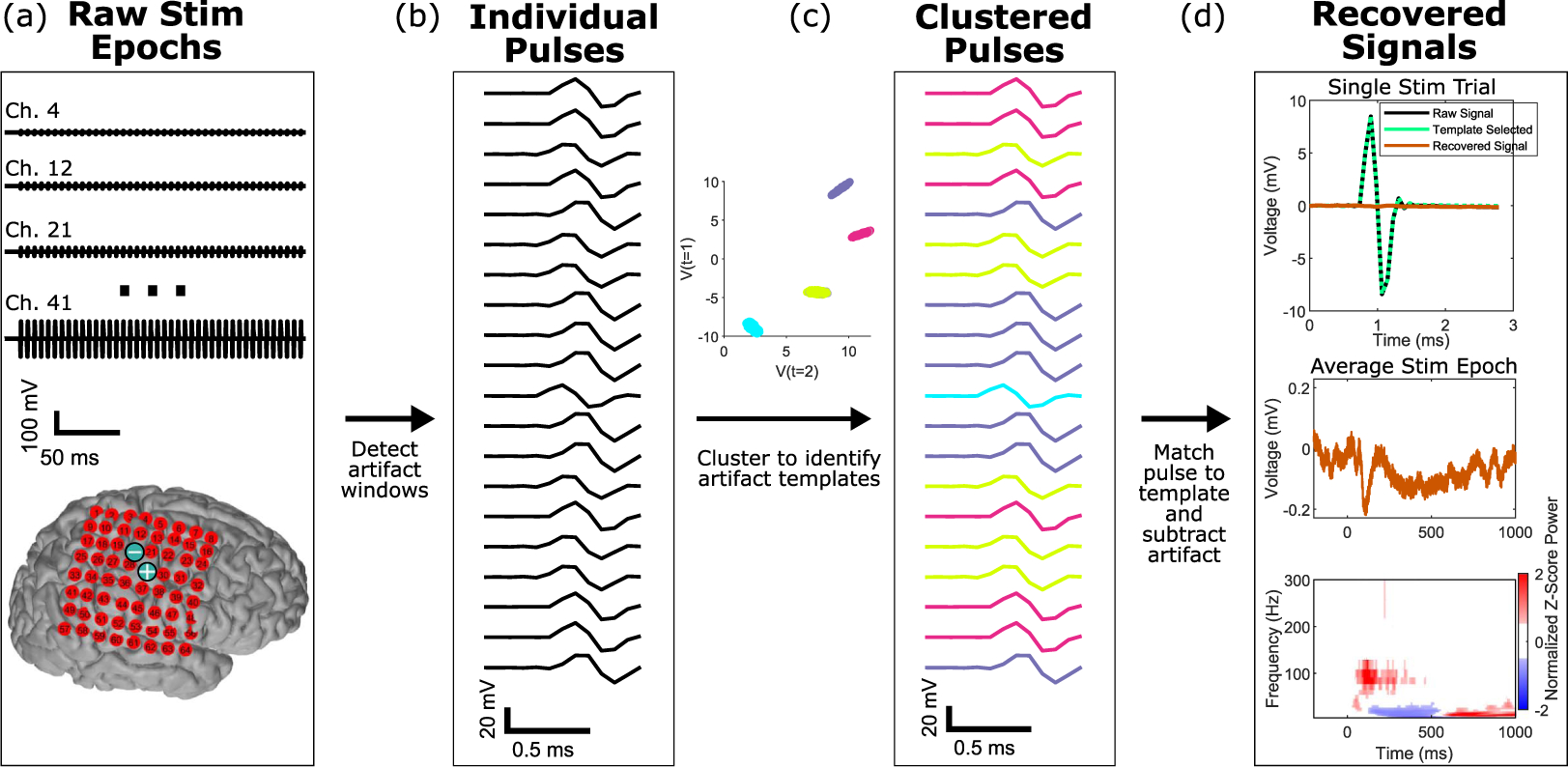
Schematic overview of our method for signal recovery with stimulation artifacts. (a) Raw stimulation signal epochs (time *×* channel *×* epoch) are recorded across an array of electrodes, as shown on a cortical reconstruction of one patient. The two electrode locations indicated by blue ⊕ and ⊖ signs were the sites of the electrical stimulation. These are the input for our algorithm. (b) Individual pulses are identified and extracted within each of these stimulation epoch time periods across all the channels in the array. A small random subset are visualized here. (c) An unsupervised hierarchical density-based clustering technique (HDBSCAN) is used to cluster the individual pulses. Each pulse is colored by the artifact template to which it clustered.(d) Signals are recovered by subtraction of the closest artifact template for each pulse. Subsequent analyses can then be performed directly on the output signals, which are the same size as the input data.

**Figure 2.**
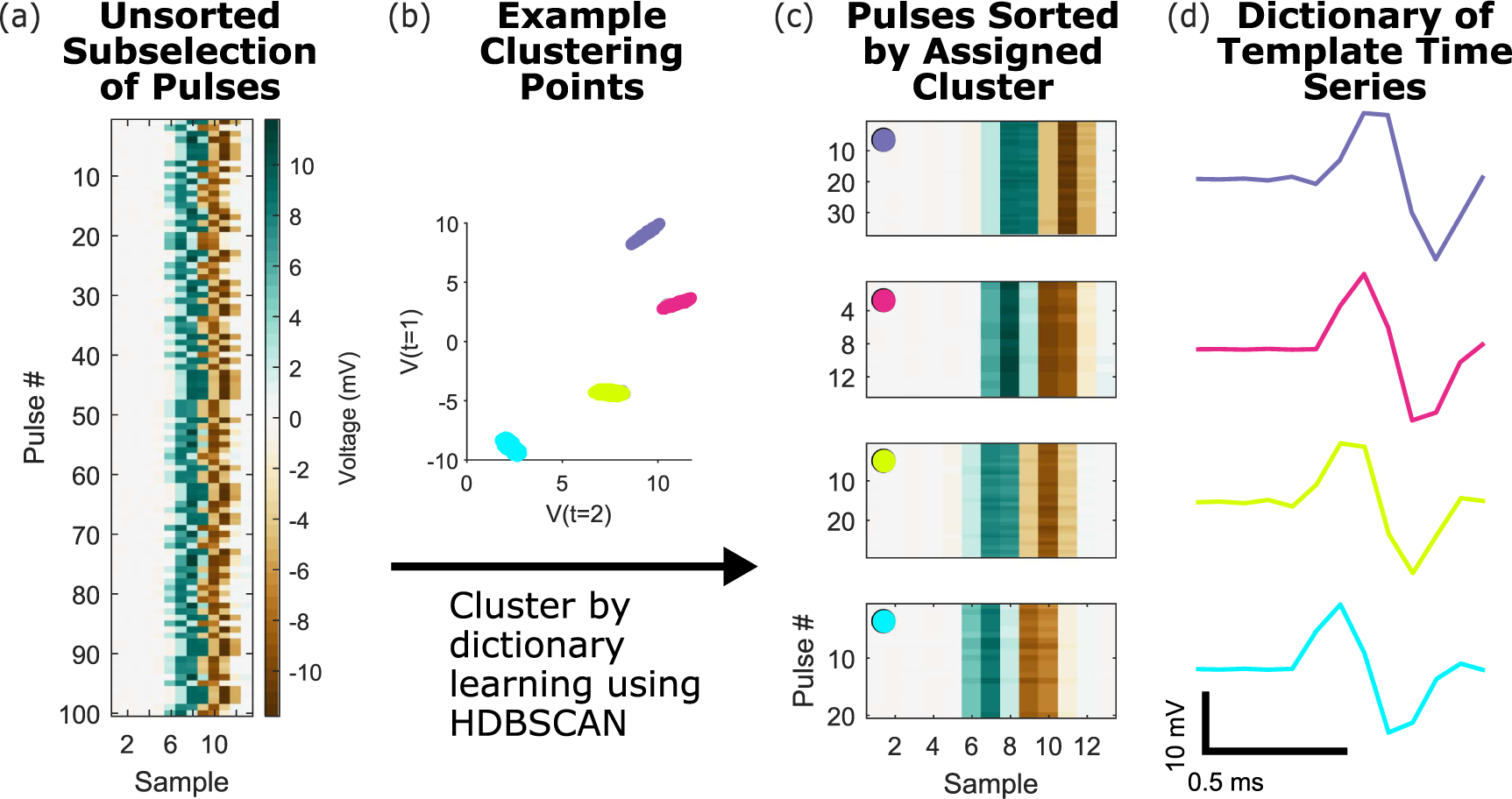
Clustering, dictionary learning, and template matching. (a) The input to clustering is a matrix of mean-subtracted raw voltages following artifact onset and offset detection, shown here as a heatmap for a small subset of trials, with the subset of data points within the artifact window used for clustering shown. The sampling rate for this data is 12 207 Hz. (b) Example voltages at two time features used for clustering, which are input into an HDBSCAN clustering algorithm. (c) The voltage data sorted by matched templates, color coded to match the clusters in panel (b). (d) The four extracted artifact template clusters for the raw traces in panel (a).

**Figure 3.**
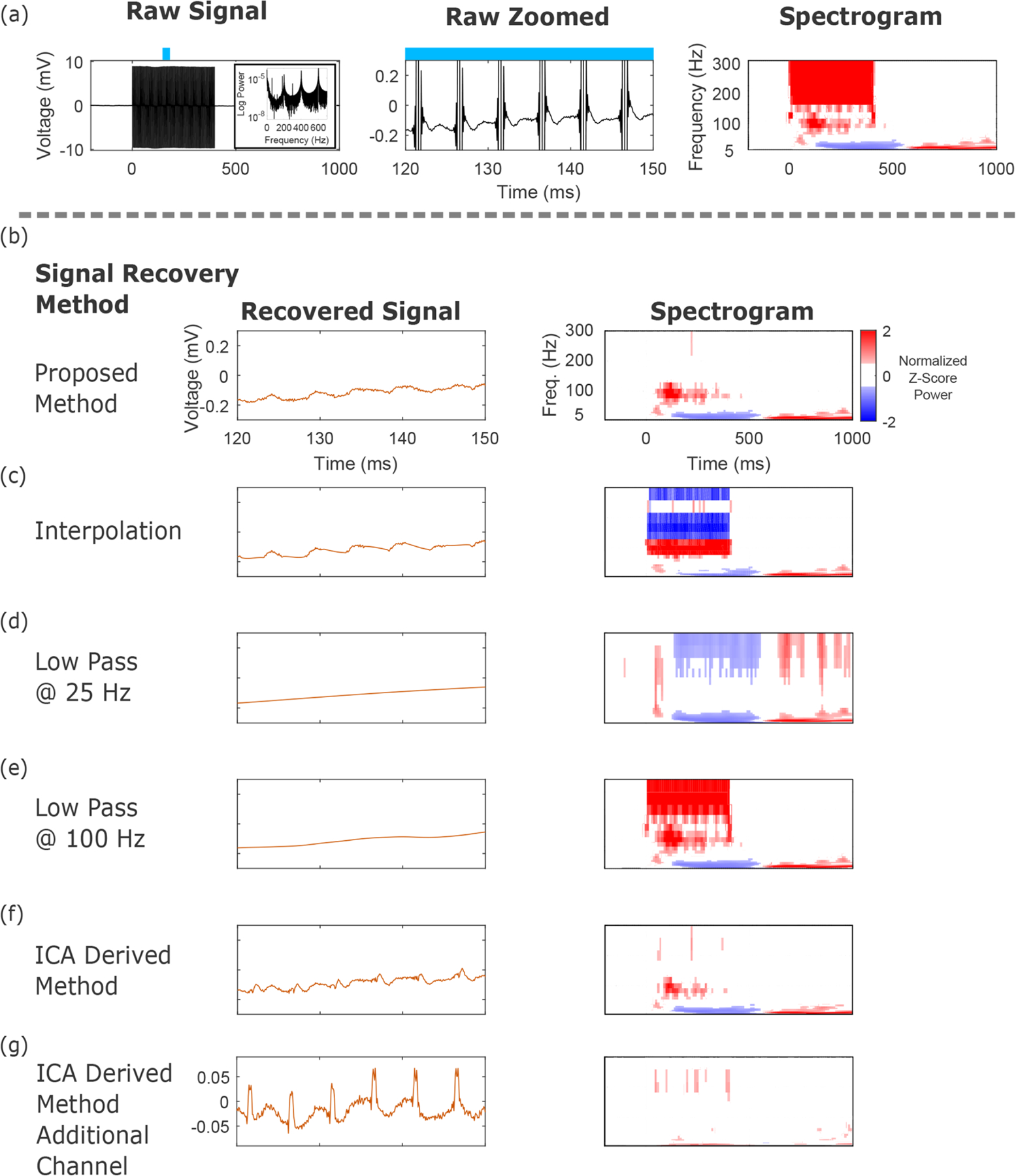
Comparisons between artifact rejection with our dictionary learning method and alternative methods as illustrated with a single channel. (a) Average raw stimulation signal across trials, from concurrent stimulation and recording. The broad spectral nature of these artifacts reveals significant overlap between spectral features of interest and the stimulation frequency. The time-frequency plot illustrates the broad spectral nature of the stimulation artifacts during the train of stimulation pulses, as well as onset and offset artifacts. (b) Signal recovery by our method has leveraged the data to account for variable artifacts in the raw voltage and timing across different channels. Our approach captures both time-series and time-frequency information (here shown averaged across all trials) well. (c) Piece-wise cubic spline interpolation locally reduces the time-domain artifact, but the time-frequency plot illustrates how large, undesirable signals have been introduced, highlighting how similar time-series traces can have significantly different spectral content. (d) Low pass filtering at 25 Hz with a 4th order acausal Butterworth filter eliminates the high frequency artifact at 200 Hz, but flattens the time-series signal and eliminates the 100 Hz activity recovered in panel (b). (e) Low pass filtering at 100 Hz fails to eliminate the high frequency artifact at 200 Hz, and flattens the time-series signal. (f) An ICA derived method that selectively removes components with a dominant 200 Hz spectral component removes the 200 Hz artifact, but also attenuates the time-varying spectral information in panel (b). (g) The same ICA derived method results in incomplete signal separation on other channels within the array, leaving large residual artifacts.

Figure 1 illustrates the overall algorithm; each step in the pipeline is elaborated in the subsequent sections. Briefly, raw recorded data during stimulation epochs from many channels are taken as input; these typically include trains of stimulation (figure 1(a)). Individual stimulation-evoked pulses within each of these epochs are detected (figures 1(b) and 3) and clustered using a density-based algorithm (HDB-SCAN, or (hierarchical density-based spatial clustering of applications with noise) (Campello *et al* 2013)) to learn a dictionary of artifact templates. Since this step is crucial for the success of our signal recovery, it is shown in more detail in figure 2. Next, each individual pulse is compared to this dictionary, and the closest template (figure 1(c)) is subtracted from the raw pulse. After subtraction, subsequent analyses in the time and frequency domains may be performed on the recovered signals directly (figure 1(d)).

### 3.1. Detection of artifact windows

We begin with *e* epochs of data that are each *t* time snapshots by *c* channels, so the size of the input is (*t × c × e*). Instead of relying on alignment to stimulation pulses, we detect artifact windows by leveraging timing information on the channel with the largest electrical artifact magnitude. To estimate artifact onset and offset, we use a Savitzky-Golay filter (Savitzky *et al* 1964) to reduce high frequency noise (3rd order, 7 samples, or 0.57 ms at 12 207 Hz). We have empirically found an absolute Z-score threshold of 1.5 on the epoched signal to successfully detect the onset of the artifacts, but this parameter can be modified by the user.

**Figure 4.**
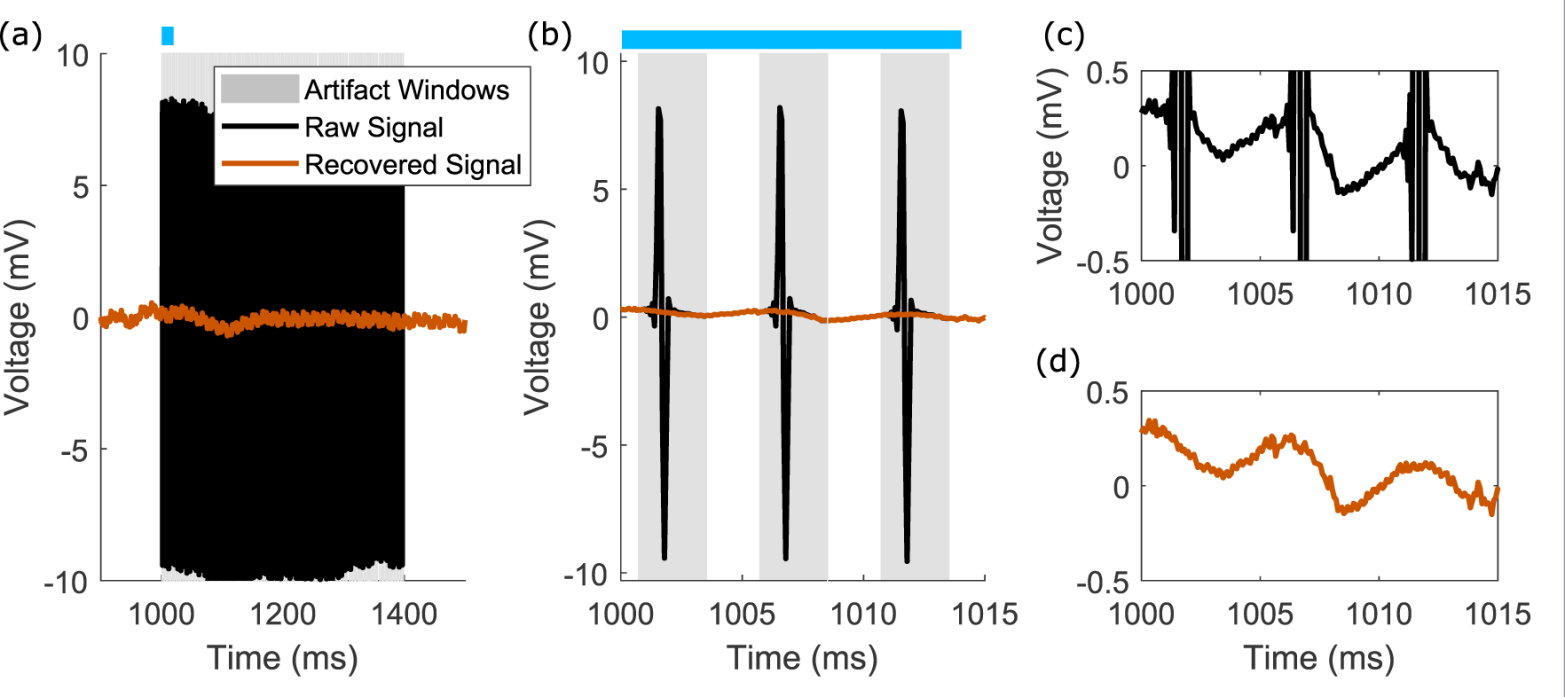
Details of the raw and recovered time-series signals. (a) The raw (black) and recovered (orange) time-series data for one epoch, with gray windows indicating the artifact windows. The channel highlighted is channel 28 in figures A1–A2. The blue bar indicates the period of time shown as zoomed in time in panel (b). (b) Zoomed-in region of panel (a), highlighting the onset and offset for each individual artifact, and the recovered signal. (c) and (d) Corresponding raw and recovered signals at a smaller voltage scale for panel (b), highlighting the preservation of signal outside of the artifact window. Signal recovery within the artifact window has no obvious discontinuities.

**Figure 5.**
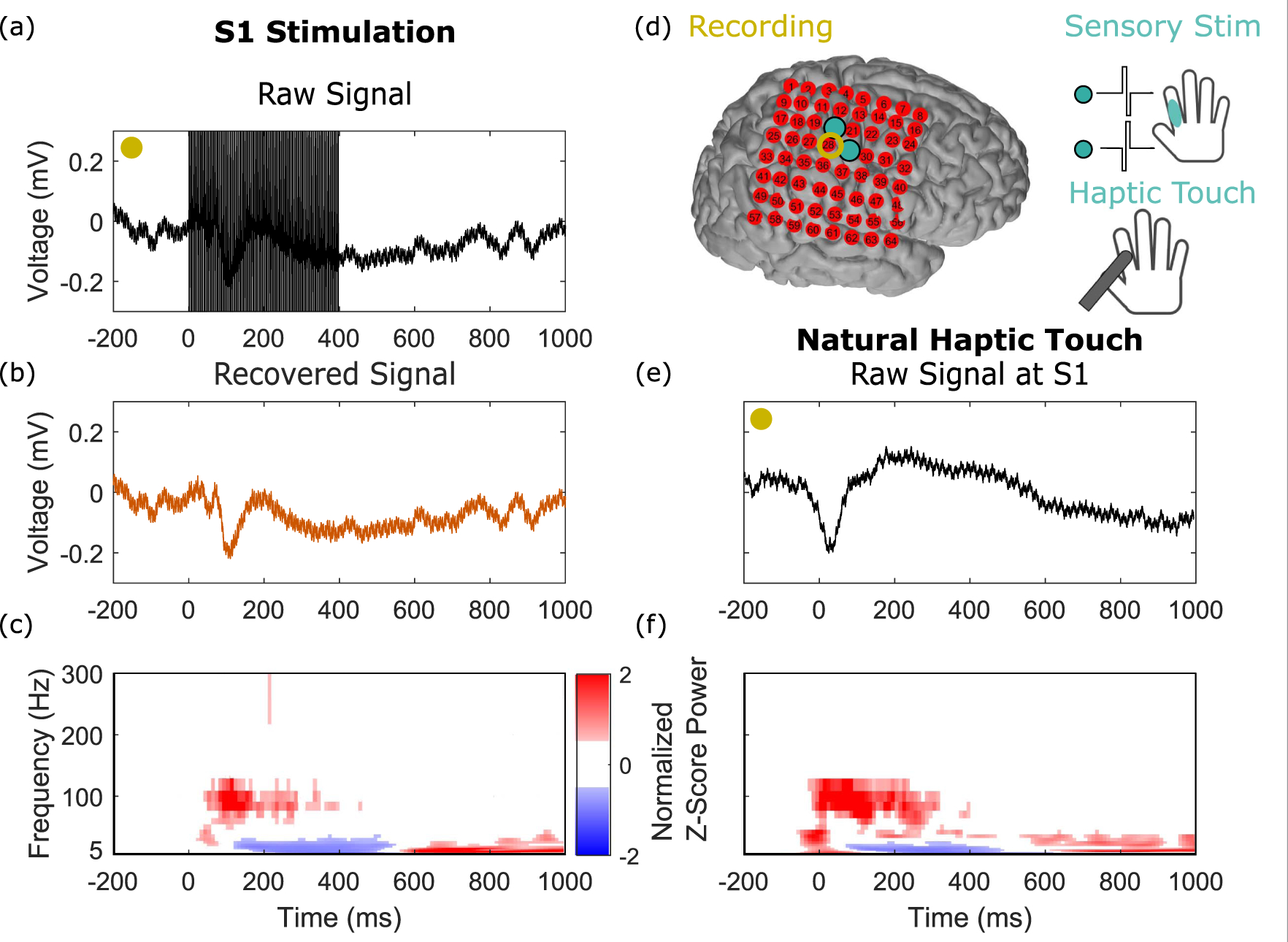
Signal recovery shows meaningful neural activity after artifact subtraction in a comparison of electrical stimulation with peripheral haptic touch. We compared responses at an electrode (yellow circle) that showed robust responses to both haptic and direct S1 stimulation. The site of the touch was matched to where the stimulation sensation was localized on the hand, as illustrated in (d). (a) The raw time-series trace, averaged over all stimulation epochs, aligned on stimulation train onset at time t = 0 ms, showing prominent stimulation artifacts (a train of pulses at 200 Hz applied for 400 ms). (b) The average of the recovered signal. (c) The time-frequency plot of the signal in panel (b). (d) The experimental paradigm. (e) and (f) The mean time-series and time-frequency plots of the haptic touch experiment aligned on touch onset, which occurs at time t = 0 ms. The small delay seen between the neural signals and t = 0, where touch onset is marked to occur, is due to previously published latencies resulting from custom electronic touch probes comprised of force-sensitive resistors (Caldwell *et al* 2019, Collins *et al* 2017).

We then define a window size (an example is below) around each stimulation pulse and align the start of these windows to extract pulses from all epochs (figures 1(b) and 2(a)). We used a time window of 0.8 ms before the detected onset of stimulation for all datasets. We subsequently used a Z-score percentage threshold on both the raw smoothed voltage and differentiated smoothed signal (we used a 75% cutoff for both of these values for figures 1–5), and used the threshold that selected the furthest time point from artifact onset for the end of each stimulation pulse. This estimate was individually computed for each pulse, in each epoch, and in each channel. An extra 1 ms was added after the end of each artifact to ensure that the entire artifact had been adequately sampled for all datasets. In this way, we transform the *t × c* × *e* input data to be a cell of size (*t*_*p*_*×c ×* (*e × p*)), where *t*_*p*_ is an index for the time window for each pulse on each channel and *p* is the number of pulses. The smoothed signal is not used further in the signal recovery pipeline.

To make these extracted individual pulses suitable for unsupervised clustering, we equalized the pulse lengths by zero-padding the end of each pulse within a channel, so that they are all the same length (figure 2(a)). In order to eliminate the baseline offset of the artifact window, a user defined number of samples is used to define a period over which an average is calculated, and this average is subtracted from the entire pulse. In this work, we have used the first 3 samples of the artifact window, as this is empirically what has worked well with our data sets, but longer time periods, as well as mean of each window could be used. These onset times, offset times, percentage thresholds, and number of samples included for offset correction are all parameters in the algorithm and are tunable by the user to the particular nuances of their data. For instance, the time window before the artifact onset and after the artifact offset can be manually changed to ensure that the entire artifact is captured, while minimizing the amount of uncontaminated neural signal that is included in artifact processing. In order to the determine the number of samples which should be included for baseline offset correction, the selected points should not extend too far before the stimulation onset to minimize the inclusion of temporally distant recorded signals. The Z-score percentage threshold for estimation of artifact offset can be modified to ensure that the end of the artifact is appropriately detected. We find that a higher percentage threshold offset resulted in better performance on our DBS datasets (see Results section). A higher percentage (e.g. 99%) would ensure the capturing of a longer duration artifact, potentially more appropriate for longer lasting exponential artifacts, while a smaller percentage threshold (75%) is potentially more appropriate for a shorter lasting artifact.

### 3.2. Comparison to recovery by alternative methods

Once each stimulus pulse has been isolated, the next step aims to reduce the artifact present within this window. The electrical artifact is typically many orders-of-magnitude larger in amplitude than the underlying neural signals (figure 3(a)), making this task particularly challenging. Additionally, the effect of the artifact extends into frequencies other than just the stimulation frequency and its harmonics. We compare our proposed method (figure 3(b)) with other commonly used strategies to recover the signal.

One strategy is an interpolation scheme that ignores the artifact itself, filling in the data between the endpoints of the stimulus artifact. As noted, variants of these have been used for electrophysiologic recordings with concurrent stimulation. Figure 3(c) shows the results of a shape-preserving piece-wise cubic interpolation scheme using data points adjacent to the stimulus artifact. Code to perform this interpolation is provided in our repository. While the recovered signal in the time domain is continuous and in the right order-of-magnitude in voltage, figure 3(c) shows that the interpolation scheme has introduced large, undesirable signals in the frequency domain.

Another common approach is low pass filtering, which as shown in figure 3(d) and (e) overly smooths the time-domain signal and either eliminates neural signal in the time-frequency domain (figure 3(d), or fails to remove the stimulation artifact (figure 3(e)). We here use filtering across the entire pulse train, rather than filtering individually detected pulses. Although the time-series signal may look smooth, the normalization to baseline may reveal spectral artifacts incompletely removed by the filtering process due to the characteristics of the filter, which include the roll-off, order, and center frequency. We used acausal, 4th order Butterworth filters for the low pass filtering. We believe the spectral components represented in the 100 Hz low pass filter case are due to the incomplete suppression of artifact components in the 100–300 Hz regime, which are revealed by the normalization to baseline.

Another approach is ICA. Here, an ICA implementation that selectively removed ICA components with very high frequency (200 Hz) spectral peaks was used to eliminate the stimulus artifacts. This implementation resulted in reasonable performance in the time-frequency domain (figure 3(f)) for the same channel as panels (a–f), but there were residual artifacts present in the time-series signal. Most importantly, other channels (e.g. figure 3(g)) had large residual artifacts. This points to the difficulty in blind source separation using ICA, even with domain-specific optimization for signals that have artifact components orders-of-magnitude greater than the neural signals of interest, that are not consistent in shape across channels.

### 3.3. Dictionary learning with unsupervised clustering to extract artifact templates

Stimulation artifacts recorded at each pulse on each channel have different waveforms. Consequently, the mean artifact waveform is a poor representation of individual artifacts. Differences in artifact morphology arise from a few different sources, including slight temporal offsets due to windowing, fine differences in timing of sampling, different stimulation parameters during and across epochs, as well as varying factors at each electrode-tissue interface. Nevertheless, there are stereotypes of artifact waveforms, and here we have implemented a data-driven strategy for learning these templates. In particular, we created a dictionary of artifact waveforms from all pulses and all epochs of each channel using unsupervised clustering.

Our algorithm uses a modified HDBSCAN algorithm for clustering (Campello *et al* 2013), which is in turn a variant of the classic DBSCAN algorithm (Ester *et al* 1996). We use a MATLAB implementation of HDBSCAN openly distributed on GitHub (Sorokin *et al* 2018). This algorithm discovers dense regions (clusters) within a noisy dataset; the algorithm determines the number of clusters that explain the data, given three key parameters that must be chosen by the user. The first parameter is the number of neighbors *k* used in the density computation; increasing this parameter restricts clusters to increasingly dense areas. The second parameter is the minimum cluster size *n*, which is to say, the minimum number of data points considered a cluster. The third parameter is the outlier score threshold *θ* = [0, 1], above which an observation is considered to be noise. For our haptic touch, variable stimulus amplitude, and DBS datasets, we specified *k* = 2, *n* = 3, and *θ* = 0.9. For our button press dataset, we used *k* = 15, *n* = 10, and *θ* = 0.95. We used euclidean distance between artifact pulses as our distance metric, but other distance metrics for clustering could be used as desired.

In order to choose appropriate values for the parameters described above, the user should first look at the raw individual pulses and decide if there are numerous visible artifact shapes, and approximately the percentage of pulses represented by each artifact shape. An overly high minimum cluster size *n* may fail to capture clusters which represent rarer artifacts. Similarly, how tightly the individual points cluster within their described clusters, and the noisiness of the data, helps inform how many neighbors *k* should be used, and the outlier score threshold *θ*.

All the pulses for each channel are clustered together. Figure 2(a) illustrates the input data matrix for the clustering; 12 features were used in the clustering, taken as 6 samples (0.5 ms) on either side of the time at which the maximum absolute value voltage occurred. The number of features can be defined by the user depending on how many informative data points are contained within a given artifact pulse. This number of features is independent of the window length, and allows for selection of the maximally separable number of points by the user for any arbitrary window length. We chose this part of the artifact to manage the dimensionality of the clustering task, focusing on the time-point features that are most informative about the separability of stimulus artifacts. Two of these time-point features are used as axes in figure 2(b), showing the clustering of artifacts into 4 distinct regions in this example.

### 3.4. Signal recovery by template matching and artifact subtraction

The mean waveforms of every cluster form the dictionary of artifact templates (figure 2(c) and (d)). The correlation of each individual pulse in the signal with each of the templates in the dictionary is calculated, and the maximally correlated template is chosen. We use correlation to minimize the impact of scaling and normalization in our template selection. Once the best template has been selected, it is subsequently scaled by the ratio of the template’s range to the individual pulse’s range. This is to adjust for slight changes in stimulation pulse amplitude across pulses. Finally, the scaled template is linearly subtracted from the time window for that pulse to recover the neural signal (figures 2(c) and (d)). We also implement for comparison an average within epoch template subtraction scheme, as well as an average across all stimulation pulses subtraction scheme, for each channel (data not shown). Under simple cases, these methods may perform as well as the more complex method detailed above, but for varying amplitude waveforms and stimulation pulses with different sampling times, our more advanced method outperforms these other approaches (see below).

### 3.5. Post-processing and visualization

**Figure 6.**
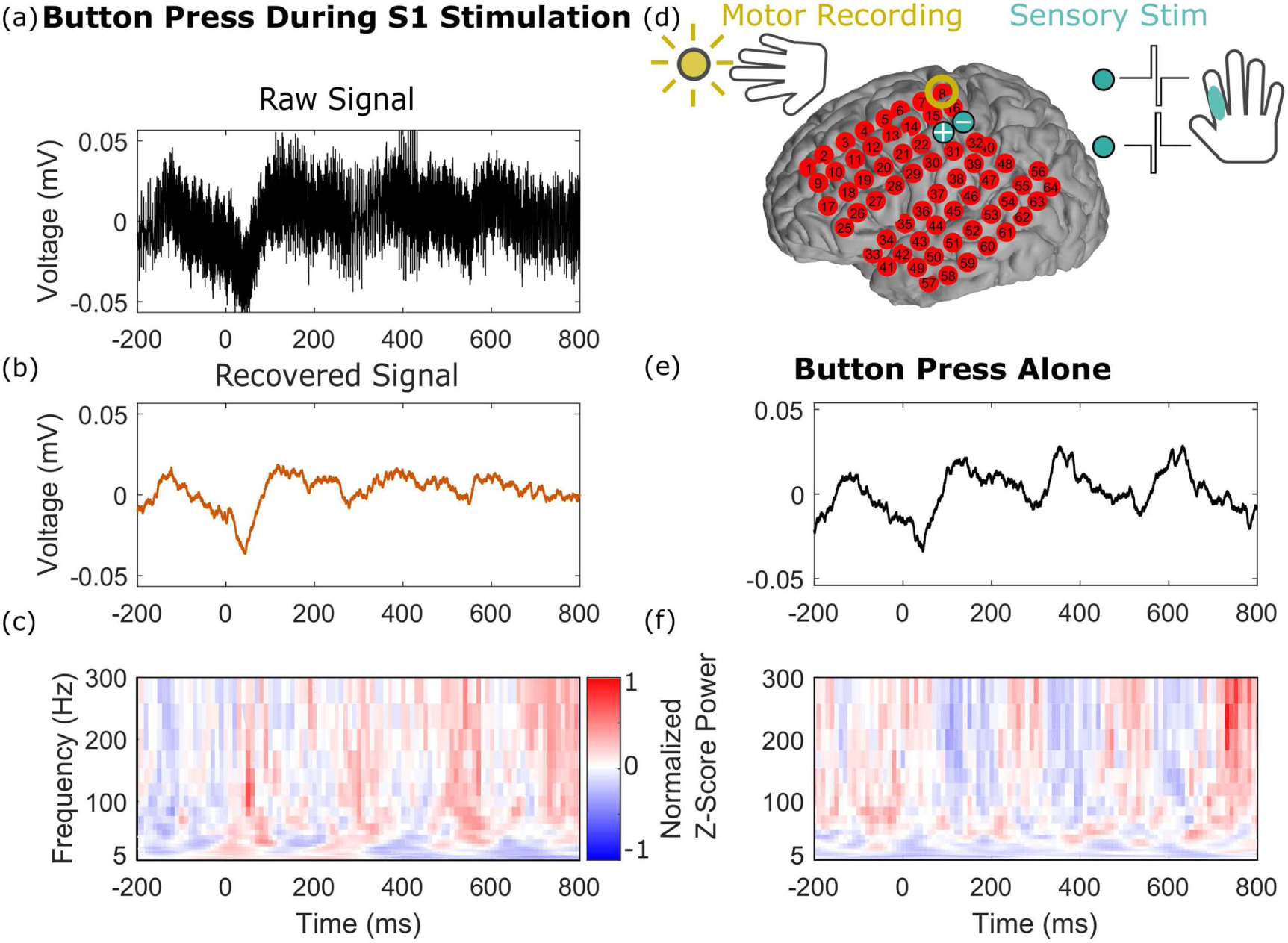
Signals recovered during a self-paced button pressing task with and without concurrent stimulation are comparable. We analyzed responses at one electrode (yellow circle in panel (d)) in motor cortex. (a) Average raw time-series trace during S1 stimulation (200 Hz trains at turquoise electrodes in (d)), zero aligned to time of button presses. (b) and (c) The average recovered signal, shown as time-series and time-frequency plots. (d) The experimental paradigm, where the subject perform a self-timed button pressing task and received electrical stimulation in S1 of the same hand on some of the trials. (e) and (f) The average time-series and time-frequency plots of the stimulation-free conditions.

Following signal recovery, we performed averaging of the epochs to obtain the average time-series visualized in this paper. For figure 6, we performed common average rereferencing against the non-stimulation channels to remove common noise. On each epoch, we use non-analytic Morlet wave-lets (Wander *et al* 2016, Bruns *et al* 2004) to calculate amplitude in time and frequency bins. We used 10 ms bins as our time resolution for the wavelet processing. We visualized the 5–300 Hz region to avoid including lower frequencies where edge effects from the wavelet processing may exist, and to focus on signals such as broadband gamma (Miller *et al* 2008). We considered broadband gamma to be an aggregate of local neuronal activity and to be more easily separated from lower frequency oscillations above 50 Hz. The time-frequency plots for the haptic touch datasets were Z-scored normalized relative to a baseline period from 800 ms to 5 ms before stimulation or before touch onset within each frequency band. In the button press datasets, the plots were Z-score normalized relative to the trial for –900 ms before button press to 900 ms after button press, as there was no consistent baseline period before each trial.

## 4. Results

**Figure A1.**
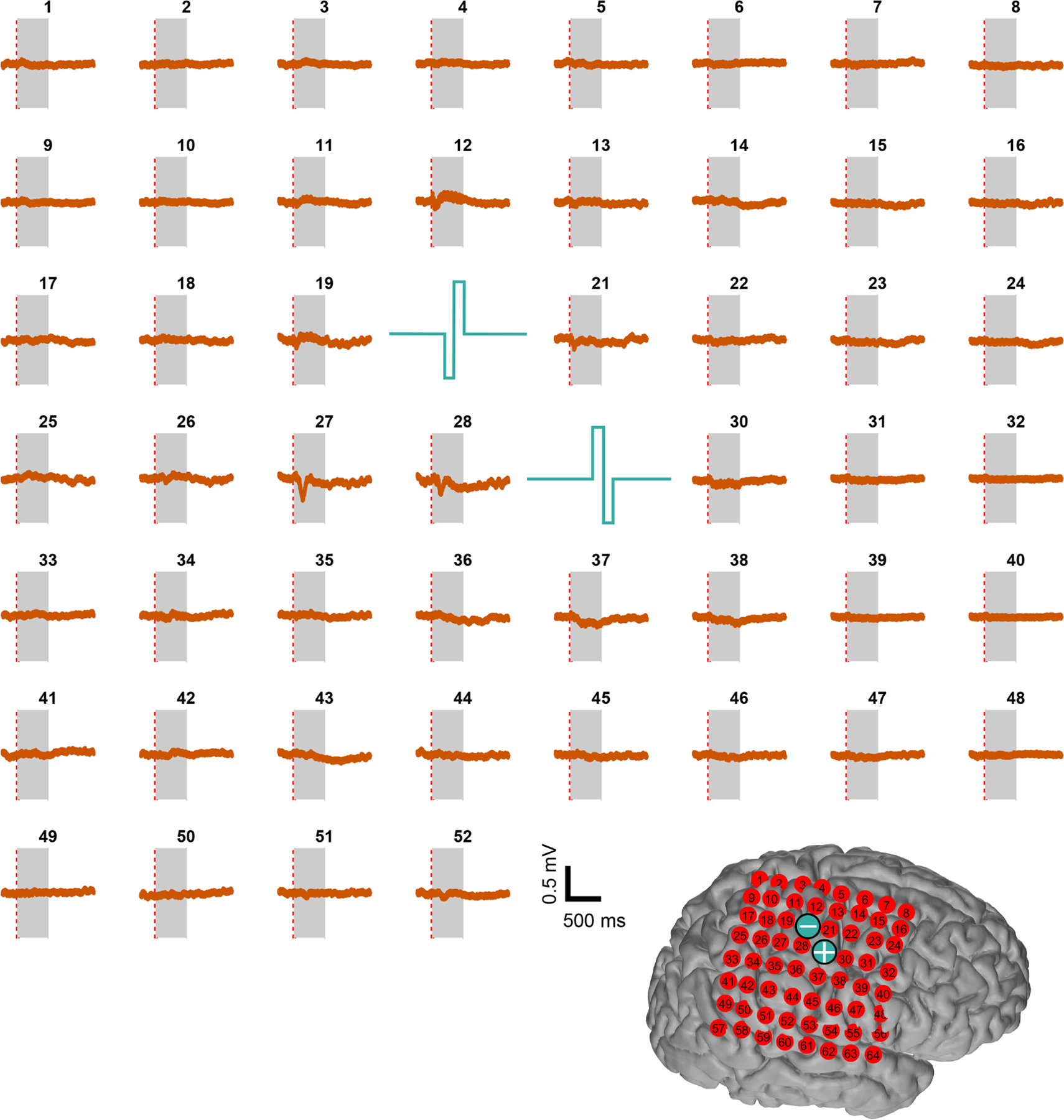
Average time-series responses following processing across cortex. Gray window highlights stimulation time period, example data file 1. Turquoise bipolar waveforms indicate the stimulation channels. (Lower right inset) Cortical reconstruction for this subject, where the sites of electrical stimulation are indicated by ⊕ and ⊖ signs.

**Figure A2.**
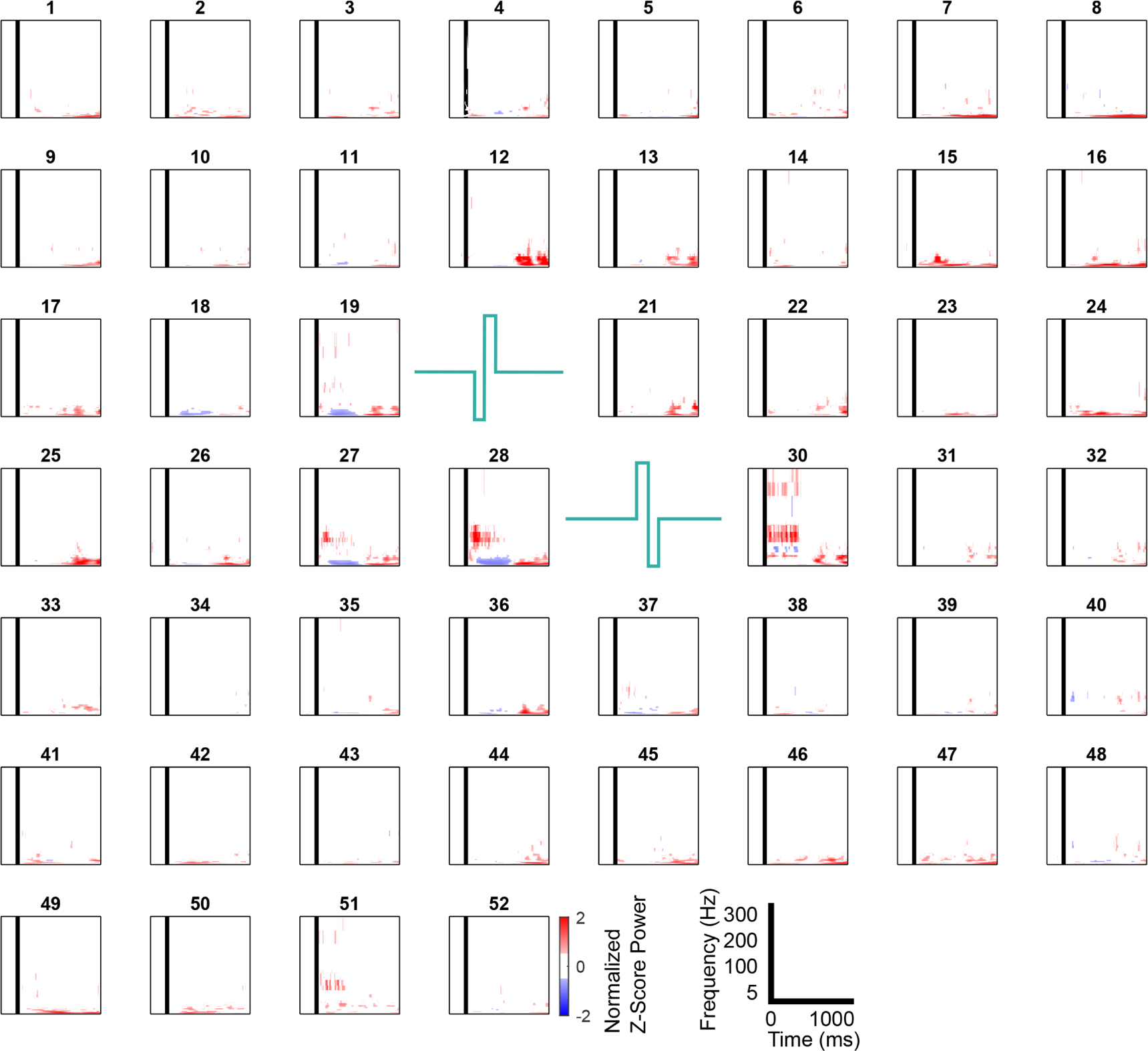
Average time-frequency responses following processing across cortex for example data file 1. Black bar indicates the beginning of the stimulation window. Turquoise bipolar waveforms indicate the stimulation channels.

Here we show that our unsupervised clustering approach to learning artifact templates is reproducible and robust, recovering underlying neural signals recorded concurrently with electrical stimulation. We demonstrate that the signals recovered in a variety of different human intracranial recording datasets are interpretable and physiologically valid. Importantly, our method protects time-points outside the stimulation epochs from processing, mitigating any adverse effects of the signal recovering algorithm on data without artifacts. Figure 4 compares the raw and recovered signals for one stimulation train epoch of one channel from an array of intracranial recordings; results from the rest of the channels for this subject and a second subject are shown in figures A1–A2.

### 4.1. Comparison between direct electrical stimulation of S1 and natural haptic touch

To demonstrate that the recovered neural activity is physiologically meaningful and valid, we highlight results comparing neural recordings from direct electrical stimulation of S1 to those from peripheral haptic touch. As illustrated in figure 5(d), the haptic touch was localized to the same region where the stimulation sensation was localized. Figures 5(a) and(e) show the raw signals recorded on the same channel, one adjacent to the stimulation electrode, where the 200 Hz stimulation train dominates the recording. Despite these large-amplitude stimulation train artifacts present in the raw signal, we observe that the overall structure of the neural response in the time domain of the recovery signal (figure 5(b)) closely resembles the raw signal from the haptic touch (figure 5(e)). The slight delay seen between the raw evoked signal and haptic touch onset at t = 0 is due to previously published latencies induced by the custom electronic touch probes comprised of force-sensitive resistors used in the experiments (Caldwell *et al* 2019, Collins *et al* 2017). Crucially, the S1 stimulation and natural haptic touch signals show strong similarities in the time-frequency domain (figures 5(c) and (f)). In addition, there is no evidence of aliasing in the spectrogram at the stimulation frequency band (200 Hz).

### 4.2. Comparison between motor activity with and without DES of S1

In a related experiment, we analyzed neural responses at an electrode that showed motor activity during a self-paced button pressing task; we compared epochs with and without concurrent stimulation of the ipsi-lateral hand S1 cortex. In figure 6, the data were aligned and averaged using the timing of the self-paced button presses. We observed a striking overlap in both the time-series and time-frequency domain between epochs with and without stimulation, as well as no evidence of residual power in the stimulation frequency band of 200 Hz. The size of the artifacts in the averaged signal before processing is smaller than in figure 5 because the epochs here are aligned not on stimulation onset, but rather on button press, resulting in the stimulation pulses being averaged out across trials.

### 4.3. Recovery with non-uniform stimulation trains

**Figure 7.**
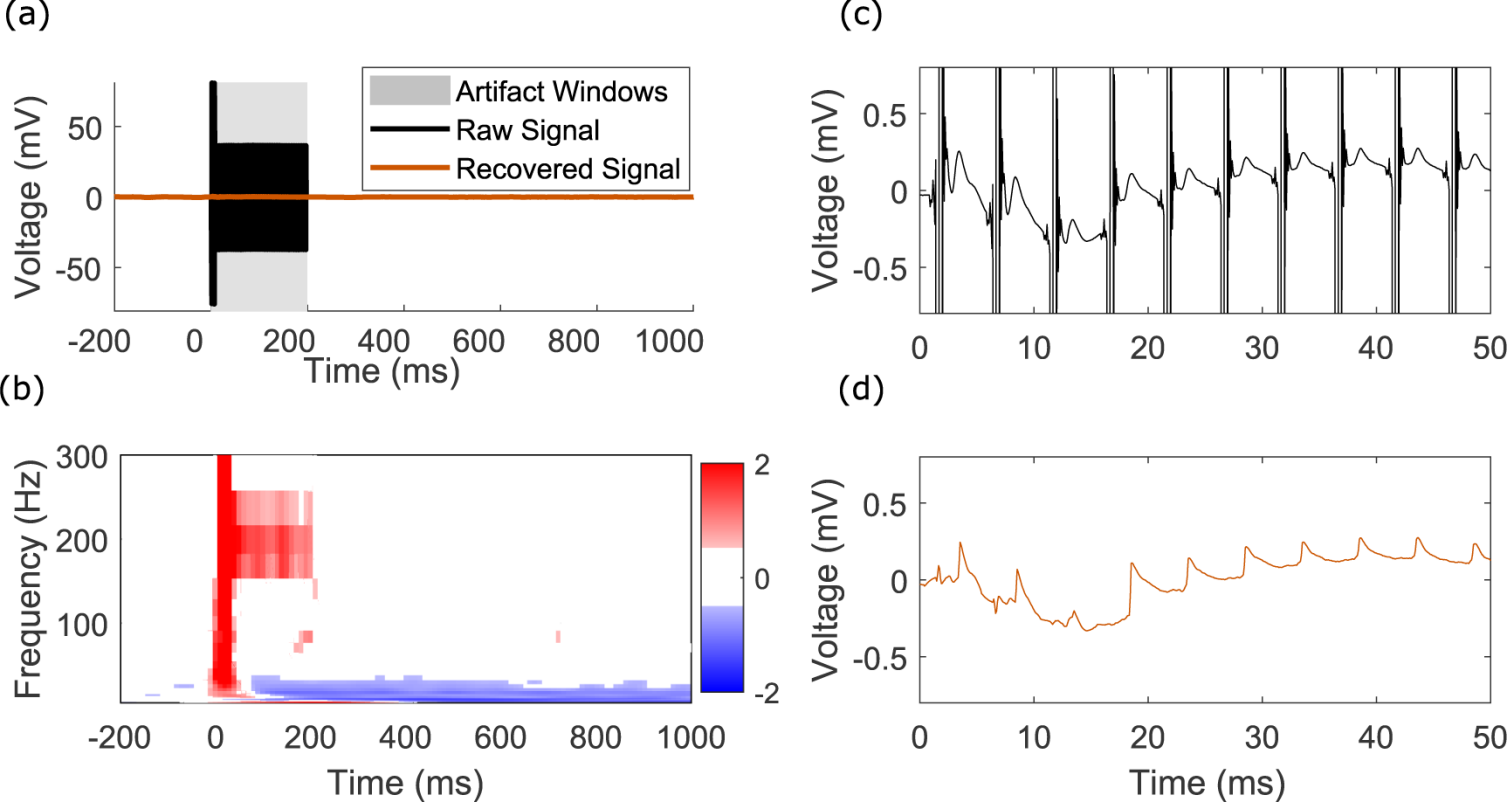
Recovery of signals from non-uniform stimulation trains and recovery of rapid evoked potentials. (a) Overlaid raw and processed signal for channel 15 for a single stimulation train epoch in figures A3–A4, highlighting two initial high amplitude pulses, followed by a train of lower amplitude pulses. Average epoched template subtraction would fail to recover the correct signal here. (b) Time-frequency plot of the recovered signal, highlighting the representation of the reproducible rapid evoked potentials in the time-frequency domain. The units are normalized Z-Score power as in other figures. (c) Zoomed-in average raw signal highlighting rapid evoked potentials following each stimulation pulse. (d) Recovered average signal highlighting the preservation of these early evoked potentials.

**Figure A3.**
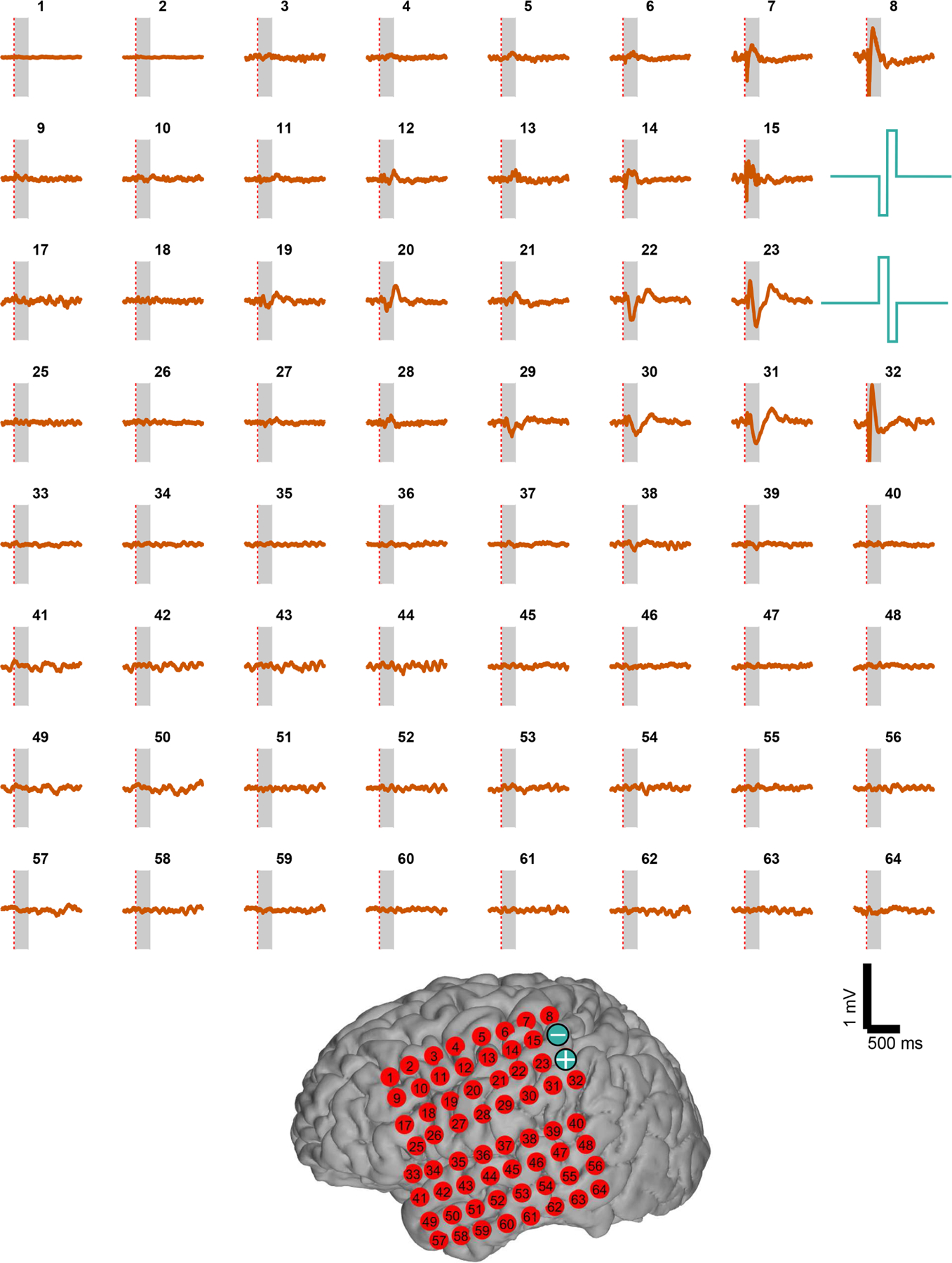
Average time-series responses following processing across cortex. Gray window highlights stimulation time period, example data file 2. Turquoise bipolar waveforms indicate the stimulation channels. (Lower inset) Cortical reconstruction for this subject, where the sites of electrical stimulation are indicated by ⊕ and ⊖ signs.

**Figure A4.**
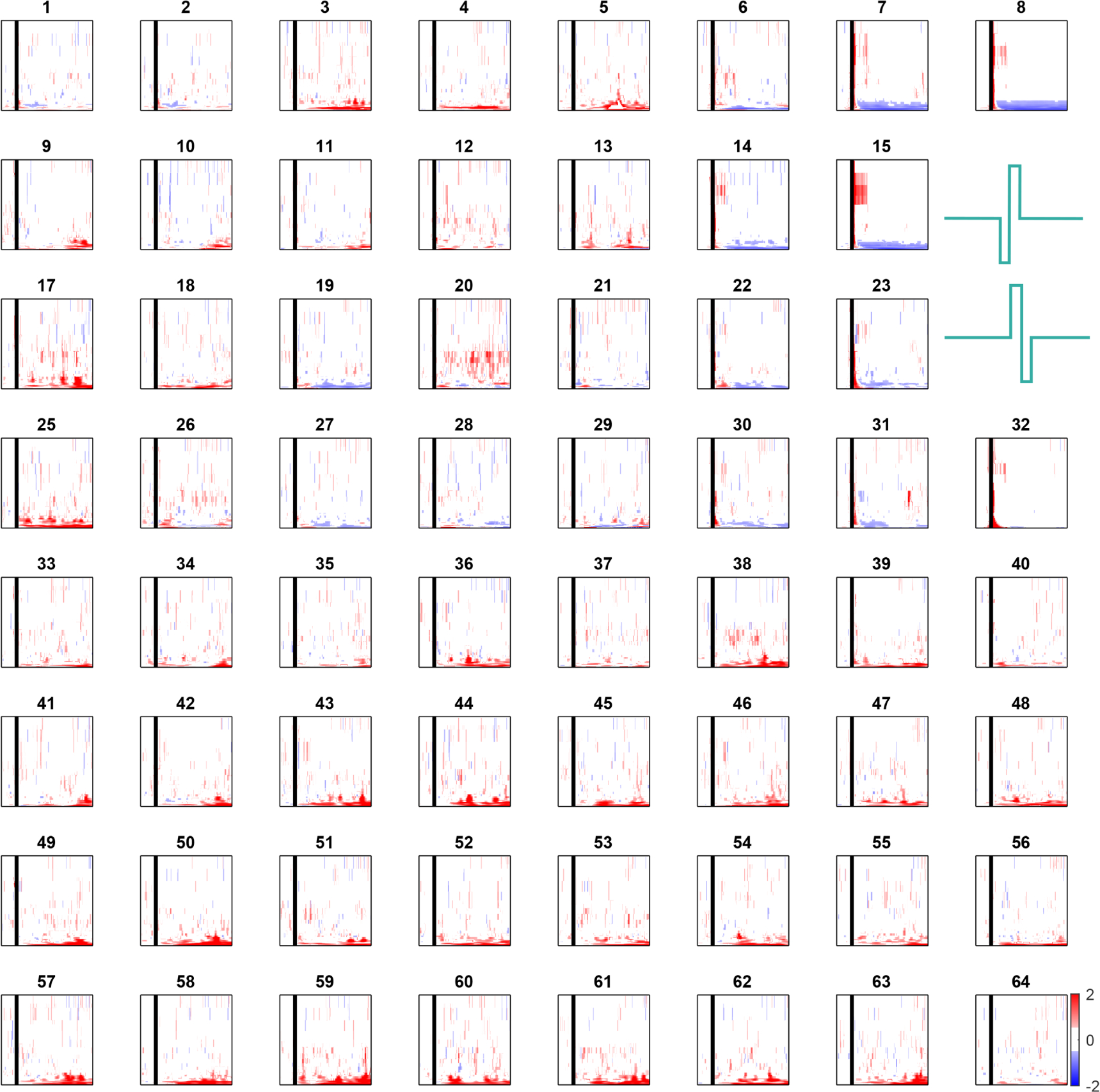
Average time-frequency responses following processing across cortex for example data file 2. Black bar indicates the beginning of the stimulation window. Turquoise bipolar waveforms indicate the stimulation channels. The x,y, and colorbar scales are the same as for figure A2.

A key feature of our algorithm is its ability to adapt to time-varying stimulation pulse amplitudes (figure 7). In this scenario, an approach where the template is computed as an average within stimulation epoch would fail. Non-uniform stimulation trains are useful because neural activity is frequently non-uniform in amplitude over time. For instance, object contact onset and offset is coded by neurons in S1 primarily through the highest firing rate during touch onset and offset, and decrease their firing during the maintenance of touch (Pei *et al* 2009). Therefore, stimulation with trains that mimic the natural firing patterns of neurons may be more effective for neuroprosthetic applications to encode contact with an object (Tabot *et al* 2013). When using a non-uniform stimulation train, we are able to recover rapid evoked potentials after the onset of each stimulation pulse which occur within 2 ms of the stimulus, which would be missed if the artifact windows were not carefully selected (figures 7(c) and (d)). In addition, the time-frequency representation of the processed signal appears to have response in the frequency band of stimulation (200 Hz), which we believe to be a real response component driven by the rapid onset evoked potentials triggered from the 200 Hz stimulation (figure 7(b)). We emphasize that we have used one set of parameters for all of the channels within this ECoG array and have been able to achieve good results across the array without detailed fine-tuning (figures A3–A4 show the time-series and time-frequency responses for the entire array).

### 4.4. Extraction of early-evoked potentials in DBS recordings and concurrent ECoG recordings with DBS stimulation

**Figure 8.**
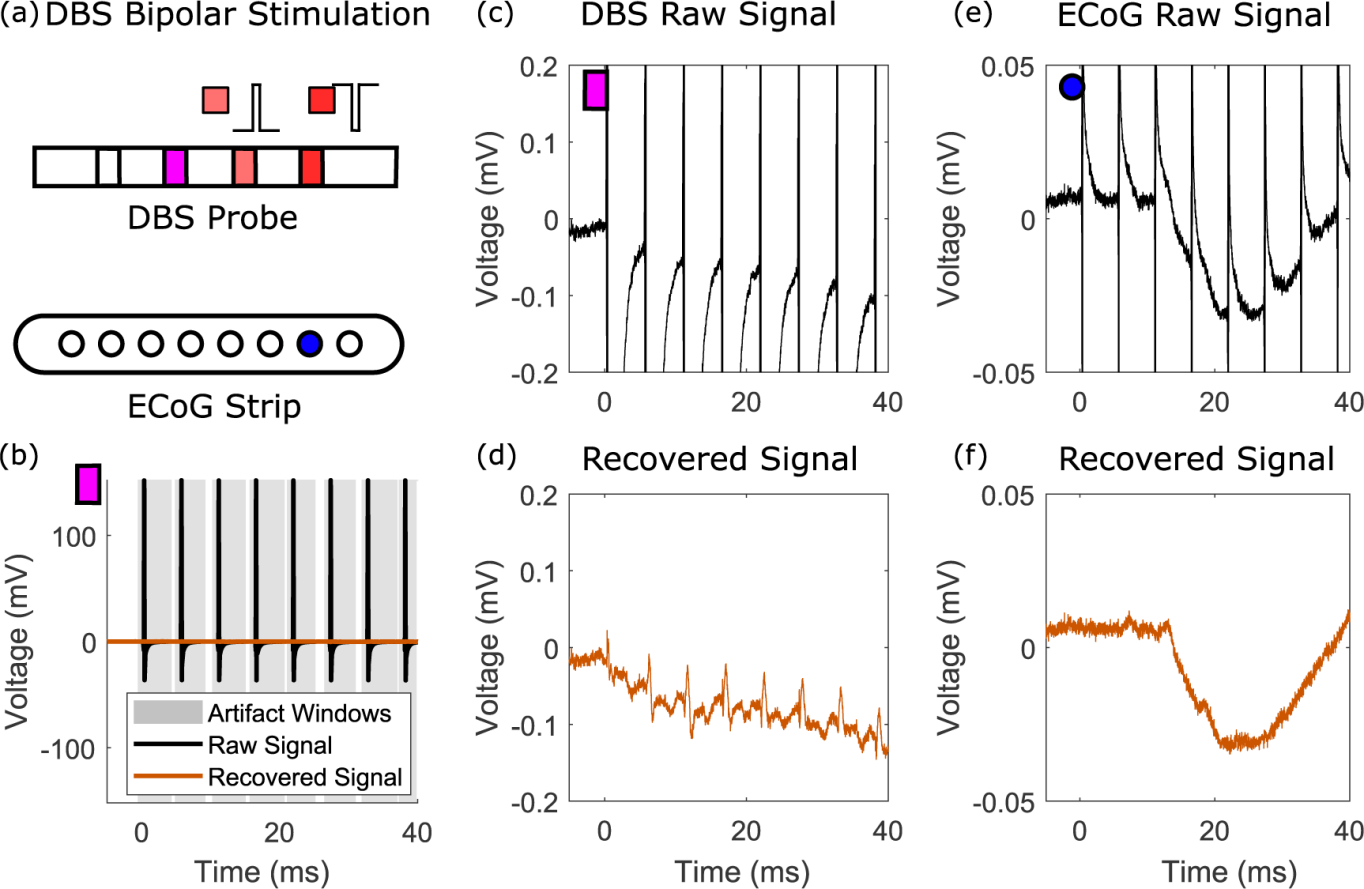
Recovery of early evoked potentials on DBS electrodes. (a) Bipolar, monophasic stimulation through DBS electrodes, with concurrent recording on the other channels. The example DBS and ECoG recordings are show by the purple rectangle and blue circle. (b) Raw and recovered example epoch, with the artifact windows highlighted. (c) Raw average signal on a DBS channel within the same probe as the stimulation electrodes. (d) Recovered average signal after template matching on the corresponding signal shown in (c), illustrating an early evoked potential. (e) Raw average signal on an ECoG electrode during stimulation through the DBS electrodes. (f) Recovered signal corresponding to (e).

In some stimulation paradigms, signals are known to exhibit long exponential recovery to baseline. We isolated early, rapid evoked potentials in deep brain stimulation (DBS) electrodes with concurrent stimulation (figure 8(a)), where Figures 8(b) and (d) show the recovered evoked potentials. In addition, using the same parameters for a concurrently recording ECoG strip yielded good performance in acquiring the resultant surface cortical activity (figure 8(f)).

### 4.5. Limitation of the algorithm

**Figure 9.**
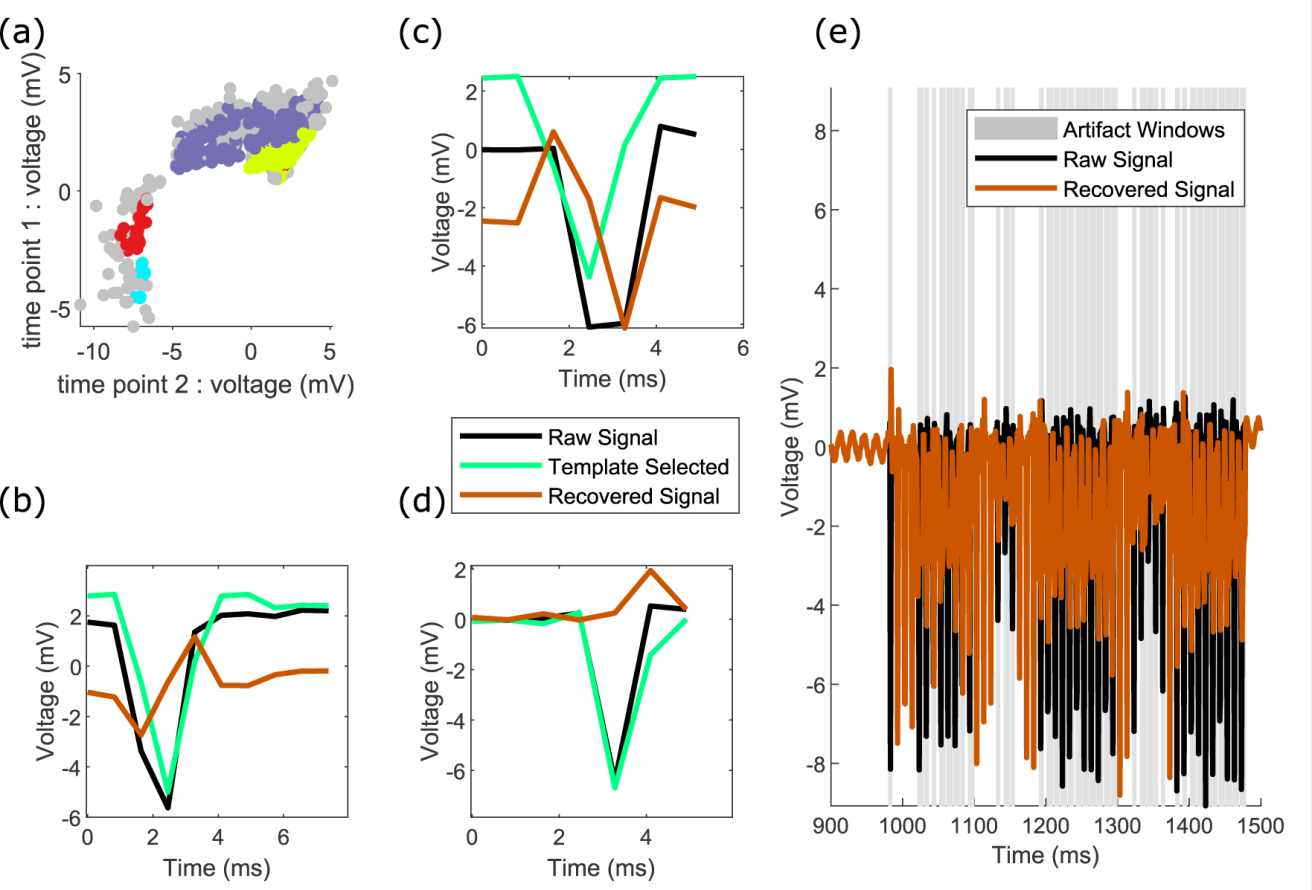
An illustration of failure to recover signal where the S1 stimulation data had been acquired at a lower sampling rate (1221 Hz) (Collins *et al* 2017). Partly due to the lower sampling rate, there were a number of failure modes as described below. (a) The density-based clustering method did not produce distinct clusters (compare to figure 2). Gray points represent individual trials which were classified as outliers and were not part of a cluster. (b) The template selected was not an ideal match and was imperfectly scaled. (c) The wrong template was selected. (d) The end of the template was not accurately calculated. (e) The result of these mismatches was unsuccessful separation of neural signal from the stimulation artifacts, shown here for an example epoch. We define unsuccessful signal recovery here as residual artifacts on the scale of the original signal, as well as no additional insight on the underlying neural activity.

Despite meaningful signal recovery in a number of circumstances, there are conditions under which the algorithms presented here are unable to recover the neural signal. One example of this failure is shown in figure 9, which shows a dataset acquired at a lower sampling rate (1221 Hz) as previously described in (Collins *et al* 2017). In particular, when the stimulation waveforms are not well resolved, such as when the signal is under-sampled, our algorithm performs poorly in discovering clusters and artifact templates (figure 9(a)). Since the template learning procedure failed, our method was unsuccessful in recovering the neural signals (figure 9(e)). We define unsuccessful signal recovery here as residual artifacts on the scale of the original signal, as well as no additional insight to the underlying neural activity (figure 9(e)).

## 5. Discussion

Analyzing signals acquired with concurrent exogenous stimulation and recording is complicated, in large part because both the physics of direct electrical stimulation and subsequent neural responses are not well understood (Borchers *et al* 2012). Therefore, there is no true ground truth on which to train and evaluate signal recovery algorithms. Previous work have made use of synthetic datasets to validate signal recovery. While simulations offer the advantage of knowing precisely what ought to be recovered, synthetic data do not accurately recapitulate key features of real data (Yang *et al* 2018). Artifacts can be different across individual channels and over time during the recordings, and may not be fully described by simple analytic models. For instance, past work has assumed constant voltage stimulation, where in our experiments we use constant current stimulation (Trebaul *et al* 2016). Beyond a simple RC circuit for the electrode-tissue interface, there are additional considerations from Faradaic and non-Faradaic reactions (Merrill *et al* 2005).

Therefore, we highlight here examples with meaningful recovery of biologically interpretable signals as the goal. This pattern of cortical responses during overt motor control is a well established phenomenon, having been observed throughout the systems electrophysiological literature (Miller *et al* 2007), and the cyclic nature of the pattern observed is illustrative of the rapid, self timed nature of the task for this subject. Similarly, the overlap in the time-series and time-frequency plots in figure 5 supports that we have extracted biologically grounded signals from our stimulation case.

### 5.1. Comparisons to commonly used signal processing techniques

Signals that are similar in the time domain may be quite different in the frequency domain (figure 3). This discrepancy points to the importance of examination of the data in both the time and frequency domains following processing. Simple filtering approaches can either overly attenuate the frequency components of interest (figure 3(d)) or fail to eliminate artifact components in the frequency domain (figure 3(e)). Modal decomposition techniques, such as our ICA implementation in figure 3(f, g), can exhibit superior performance to interpolation and filtering approaches, but it is difficult to ensure satisfactory performance across the entire array of electrodes (figure 3(g)). Methods such as ICA rely on common temporal structures across various recording sites for optimal performance, while our method considers channels independently in the detection, clustering, and subtraction process, which enables it to work well on datasets where there are small numbers of recording electrodes within an array (strip electrodes), as well as larger numbers (grid electrodes). Other advanced modal decomposition techniques as discussed in the introduction (Mena *et al* 2017, O’Shea *et al* 2017), make different assumptions about the nature of the neural and artifact components of the data. For instance, Mena *et al* 2017 use knowledge about the electrical signals known in advance from spontaneous activity to guide artifact suppression, while O’Shea *et al* 2017 utilize information regarding the spatial spread of the artifact and neural signals to separate the two. Future work could explore the possibility of extending these approaches to intracranial human datasets.

In our time-frequency plots (figures A2, A4), we do not see aliased power at frequencies lower than the stimulation frequency, which is a potential issue with template approaches on epoched data without sufficient oversampling and artifact removal (Lio *et al* 2018). The extent of our oversampling is visible in figure 1, where the maximum and minimum values for each of the stimuli within a stimulation epoch do not vary greatly. We acknowledge that our approach is not appropriate for all instances of human electrophysiologic data.

### 5.2. Comparison to other template approaches

There has been much progress in the past decades with other template subtraction approaches (Hashimoto *et al* 2002), and the methods described here are an extension of these ideas. Our algorithm leverages information across many channels at once, on a pulse-wise, channel-wise basis, by determining the likely window of the artifacts, and by clustering templates even in the presence of noisy outlier trials. The results shown in figure 4 may be captured by a simpler approach where templates are computed as means within each epoch. However, in the case of more complex waveforms with varying parameters within a stimulation train (figure 7), an average template during a train would be unsuccessful in recovering the underlying neural signal. Even so, our use of unsupervised clustering for template learning inherits some of the limitations of density-based methods (Aghabozorgi *et al* 2015). Specifically, artifacts with many points require clustering in higher dimensions, which is both less computationally efficient and requires more densely sampled data.

### 5.3. Physical basis of the structure in the data

Within each single-pulse artifact, there are physiologic, thermal, and electronic noise contributions. These are likely reflected in the heterogeneity seen within the learned clusters (figure 2b). The variance around the clusters suggests these variables are continuously distributed around discrete learned clusters. As our ideal stimulation waveforms are square pulses, even with a high sampling rate, it is very difficult if not impossible to optimally oversample the resulting waveforms, depending on the actual slew rate of the stimulator and shape of the delivered waveform. The discrete clusters in our datasets are likely due to the partial synchronization of the recording and stimulation hardware, where the recording hardware is operating at a lower frequency than the stimulator and overall clock of the device. In the event of perfectly synchronized hardware, and optimally over-sampled data, we would expect there to a single cluster, with continuously distributed variance around the center of the cluster. One of the advantages of our method is that it automatically discovers unique, independent clusters on each electrode, which makes it appropriate for events where there multiple or single clusters, which could be different on different electrodes.

Stimulation artifacts, and more so trains of stimuli, are multifactorial, and consist not only of single-pulse artifacts, but also onset and offset artifacts at the beginning and end of stimulation trains, the buildup of capacitive charge during a train of stimulation. Our work here primarily addresses the issue of consistent single pulse artifacts. The other issues are outstanding issues in the field of artifact processing, and will require future work to further eliminate the influences of these factors on the neural data. Advanced modal decomposition techniques, as well as in-vitro characterization of the precise set of electrodes and stimulation parameters used, may allow for further insight into the nature of these other artifacts and aid in their removal.

### 5.4. Practical recommendations

The code provided includes default parameters that perform reasonably well for the included datasets. A key point is that all channels within a given data-set were processed with the same parameters. Better performance could be achieved with tuning of parameters for individual or different groups of channels. Future work could include an optimized parameter search for the various parameters utilized in our approach such as the minimum number of neighboring points for a cluster or the cutoff threshold for being labeled as noise, with the use of an objective function to select the set of parameters that results in the most representative recovery of neural signal. This remains a challenging problem due to the difficulties in knowing what the best objective function and ground truth signal for optimal. Without adequate oversampling, alternative approaches using upsampling (Sun *et al* 2016) may be appropriate, or in a worst case, interpolation could be performed to estimate the time course of lower frequency signals. Where residual low frequency aliasing occurs, a Hampel filter, which has been successfully used for concurrent DBS/EEG stimulation and recording, could be used (Lio *et al* 2018). The use of simulated artifacts added to synthetic or recorded neural data could be an additional method of algorithm validation, as in Sellers *et al* 2019, and parameter exploration in the future.

In our experience, 200 Hz stimulation trains with pulse widths of 200 *μs*, sampled at 1221 Hz (figure 9) were too under-sampled for use with our algorithm. In our work here, we have focused on stimulation frequencies of 185 and 200 Hz due to the nature of the experiments from which this data was acquired. Future work could extend the approaches here to validate its performance on a wider range of stimulation frequencies. In our work, we have focused on adjacent, bipolar pairs of stimulating electrodes. Pairs of electrodes which are not adjacent may have different performance upon processing with our algorithm, and this could be addressed by future work. Our work focused primarily on recording and stimulation through grid macro ECoG arrays, with an additional DBS dataset containing ECoG strip and DBS lead data.

### 5.5. Future extensions

The analysis of electrical recordings with concurrent stimulation may benefit from future developments driven by modern methods in simulation and machine learning. A combination of model-based and data driven approaches would help construct a more principled set of templates to separate neural activity from electrical artifacts. Deep learning, which has begun to find applications in signal processing (Yu *et al* 2011) and ECoG processing (Wang *et al* 2018), could also benefit the artifact processing community if enough training data were available. Future work could extend these approaches to penetrating depth electrodes and micro ECoG arrays, as we would expect the artifact signals and neural data to behave similarly for processing.

## 6. Conclusions

Electrical stimulation applied concurrently with recording results in signals that contain both neural activity and electrical artifacts. Here we developed a novel algorithm that automatically detects electrical artifacts across many channels of recording, constructs a dictionary of learned templates using unsupervised clustering, and performs pattern matching to best extract the underlying neural activity. Rather than evaluate our method on synthetic data, we demonstrated the efficacy of our approach on real human stimulation data in both electrocorticographic (ECoG) arrays and deep brain stimulation (DBS) recordings. Further, we showed that the signals recovered have physiologically meaningful neural signatures in two datasets, providing good ecological validity for our method. In the first dataset, we showed that responses to stimulation of primary somatosensory (S1) and natural peripheral touch elicited similar responses. In the second, we showed that motor cortex activity in a button pressing task was similar with and without S1 stimulation. All of the code we developed and data sets we used have been made openly and publicly available.

## 7. Code and data availability

Full MATLAB code and a link to the data in the paper is available at https://github.com/davidjuliancaldwell/artifactRejection.

We hope these datasets may serve as benchmark datasets for future algorithm development and testing.

## Appendix A. Array-wide processed signals

To better illustrate the performance of our algorithm across all channels processed, we highlight the method’s performance on two subjects, where we used one set of parameters for all of the channels. Parameter tuning for subsets of channels would result in better performance of the algorithm, but we seek to highlight that with one global set of parameters reasonable performance is achieved.
